# Supplementary material for: Molecular analysis of a public cross-neutralizing antibody response to SARS-CoV-2
Source: Cell Rep. 2022 Oct 27;41(7):111650. doi: 10.1016/j.celrep.2022.111650 (PMC9606039; doi:10.1016/j.celrep.2022.111650)
Supplement: Document S1. Figures S1 and S2 [file mmc1.pdf]

Cell Reports, Volume *41*

Supplemental information

Molecular analysis of a public cross-neutralizing antibody response to SARS-CoV-2

Meng Yuan, Yiquan Wang, Huibin Lv, Timothy J.C. Tan, Ian A. Wilson, and Nicholas C. Wu

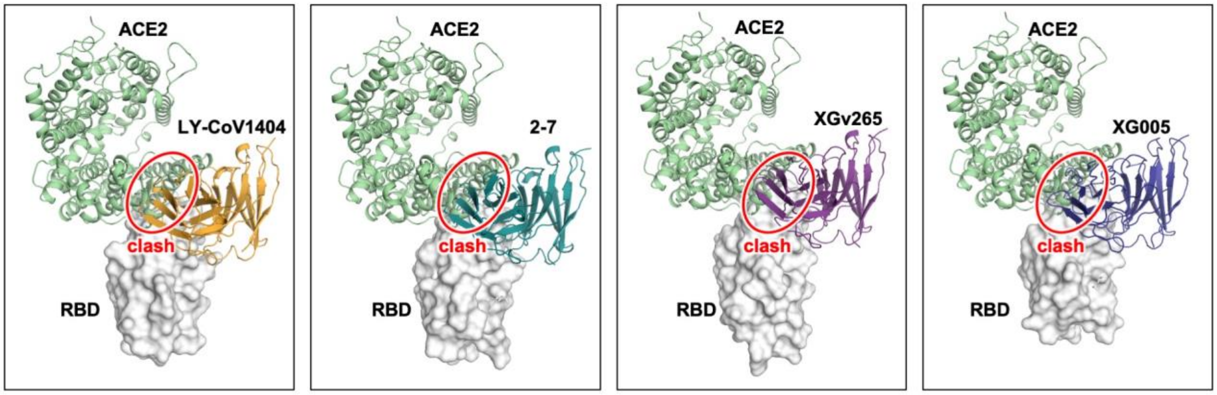

Figure S1. IGHV2-5/IGLV2-14 antibodies would clash with ACE2 binding, Related to Figure 1. Structures of antibody/RBD complexes are superimposed onto the RBD/ACE2 complex structure (PDB 6M0J)[42]. RBD is represented by a white surface. ACE2 is shown as green cartoon. LY-CoV1404: PDB 7MMO[7]. 2-7: PDB 7LSS[22]. XGv265: PDB 7WEE[19]. XG005: PDB 7V26[21].

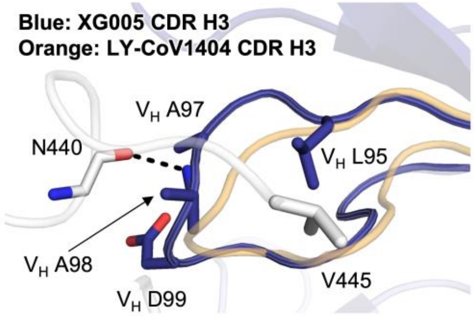

Figure S2. Interactions between RBD and CDR H3 of XG005, Related to Figure 3. A cryoEM structure of SARS-CoV-2 spike protein in complex with XG005 (PDB 7V26) that was reported in a previous study[21] is shown. A hydrogen bond between XG005 and the RBD is represented by a black dashed line. The CDR H3 of LY-CoV1404 (PDB 7MMO)[7] is also shown here as a transparent orange cartoon to demonstrate the relative positions of the CDR H3 loops from these two antibodies after superimposition.
