# Supplementary material for: Molecular analysis of a public cross-neutralizing antibody response to SARS-CoV-2
Source: Cell Rep. 2022 Oct 27;41(7):111650. doi: 10.1016/j.celrep.2022.111650 (PMC9606039; doi:10.1016/j.celrep.2022.111650)
Supplement: Document S2. Article plus supplemental information [file mmc4.pdf]

Cell Reports

Report

Molecular analysis of a public cross-neutralizing antibody response to SARS-CoV-2

Graphical abstract

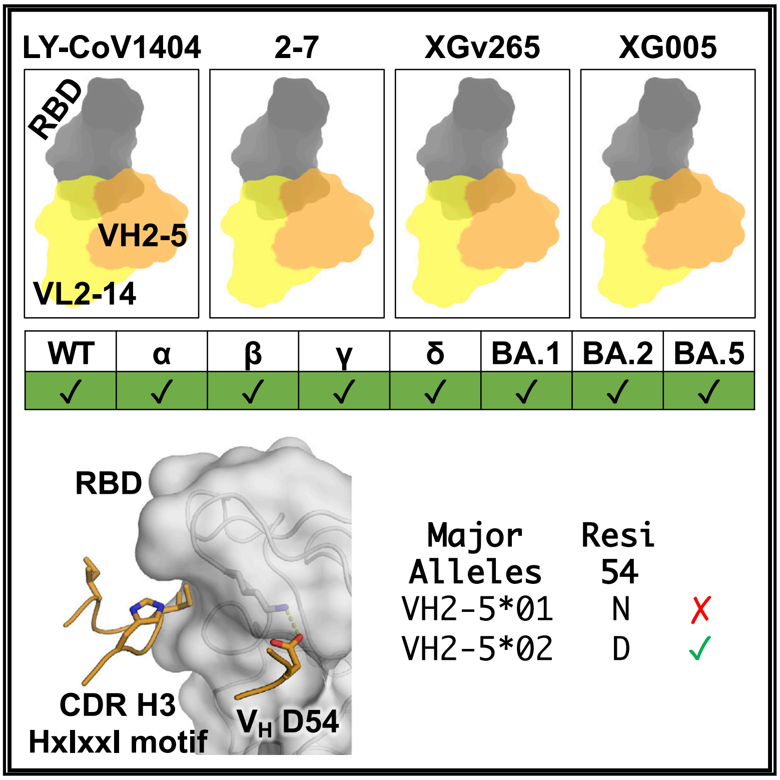

Authors

Meng Yuan, Yiquan Wang, Huibin Lv, Timothy J.C. Tan, Ian A. Wilson, Nicholas C. Wu

Correspondence

nicwu@illinois.edu

In brief

Through literature mining, Yuan et al. find that many IGHV2-5/IGLV2-14 RBD antibodies can broadly neutralize SARS-CoV-2 VOCs. Their CDR H3 sequences have a length preference and a conserved HxIxxI motif. These antibodies also have a strong allelic preference toward IGHV2-5*02 due to an allelic polymorphism at residue 54.

Highlights

- IGHV2-5/IGLV2-14 encode a public antibody response to SARS-CoV-2 RBD
- IGHV2-5/IGLV2-14 RBD antibodies broadly neutralize variants of concern
- IGHV2-5/IGLV2-14 RBD antibodies have a conserved HxIxxI motif in CDR H3
- A strong allelic preference of IGHV2-5 is essential for RBD binding

Yuan et al., 2022, Cell Reports *41*, 111650
November 15, 2022 © 2022 The Author(s).
https://doi.org/10.1016/j.celrep.2022.111650

Cell Reports

CellPress
OPEN ACCESS

Report

Molecular analysis of a public cross-neutralizing antibody response to SARS-CoV-2

Meng Yuan,[1] Yiquan Wang,[2] Huibin Lv,[2,3] Timothy J.C. Tan,[4] Ian A. Wilson,[1,5] and Nicholas C. Wu[2,4,6,7,8,*]
[1]Department of Integrative Structural and Computational Biology, The Scripps Research Institute, La Jolla, CA 92037, USA
[2]Department of Biochemistry, University of Illinois at Urbana-Champaign, Urbana, IL 61801, USA
[3]HKU-Pasteur Research Pole, School of Public Health, Li Ka Shing Faculty of Medicine, The University of Hong Kong, Hong Kong SAR, China
[4]Center for Biophysics and Quantitative Biology, University of Illinois at Urbana-Champaign, Urbana, IL 61801, USA
[5]The Skaggs Institute for Chemical Biology, The Scripps Research Institute, La Jolla, CA 92037, USA
[6]Carl R. Woese Institute for Genomic Biology, University of Illinois at Urbana-Champaign, Urbana, IL 61801, USA
[7]Carle Illinois College of Medicine, University of Illinois at Urbana-Champaign, Urbana, IL 61801, USA
[8]Lead contact
*Correspondence: nicwu@illinois.edu
https://doi.org/10.1016/j.celrep.2022.111650

SUMMARY

As severe acute respiratory syndrome coronavirus 2 (SARS-CoV-2) variants of concerns (VOCs) continue to emerge, cross-neutralizing antibody responses become key toward next-generation design of a more universal COVID-19 vaccine. By analyzing published data from the literature, we report here that the combination of germline genes IGHV2-5/IGLV2-14 represents a public antibody response to the receptor-binding domain (RBD) that potently cross-neutralizes a broad range of VOCs, including Omicron and its sub-lineages. Detailed molecular analysis shows that the complementarity-determining region H3 sequences of IGHV2-5/IGLV2-14-encoded RBD antibodies have a preferred length of 11 amino acids and a conserved HxIxxI motif. In addition, these antibodies have a strong allelic preference due to an allelic polymorphism at amino acid residue 54 of IGHV2-5, which is located at the paratope. These findings have important implications for understanding cross-neutralizing antibody responses to SARS-CoV-2 and its heterogenicity at the population level as well as the development of a universal COVID-19 vaccine.

INTRODUCTION

The effectiveness of COVID-19 vaccines has been challenged by the evolution of diverse severe acute respiratory syndrome coronavirus 2 (SARS-CoV-2) variants in the past 2 years. The recent emergence of Omicron and its sub-lineages BA.2, BA.2.12.1, BA.4, and BA.5 further highlights the urgent need for a more broadly protective vaccine. An ideal COVID-19 vaccine should elicit high titers of neutralizing antibodies that are potent against antigenically distinct variants. However, many potent neutralizing antibodies only have limited cross-reactivity for variants other than the immunizing strain. For example, a major class of antibodies to the receptor-binding domain (RBD) that are encoded by IGHV3-53/3-66 are highly potent against the ancestral Hu-1 strain, but most of them lose their activity against many of the variants.[1,2] Similarly, beta-specific antibodies can be elicited without cross-neutralizing activity against ancestral or other variants.[3] On the other hand, antibodies to S2 are typically broadly reactive but have weak neutralizing activity.[4–6] Nevertheless, a few RBD antibodies exhibit marked neutralization potency and breadth, as exemplified by those to the RBS-D epitope.[2]

One representative RBS-D antibody is LY-CoV1404 (also known as Bebtelovimab), which is a monoclonal therapeutic antibody from Eli Lilly. LY-CoV1404 is encoded by IGHV2-5/IGLV2-14 and can cross-neutralize the ancestral Hu-1 strain as well as all known variants of concern (VOCs), including Omicron and circulating sub-lineages.[7,8] In fact, the binding mode of LY-CoV1404 is identical to the cross-neutralizing antibody 2-7, which is also encoded by IGHV2-5/IGLV2-14.[9] More recently, Veesler and colleagues reported another potently cross-neutralizing antibody with similar sequences and binding mode as LY-CoV1404.[10] As IGHV2-5 was shown to be an important contributor to the cross-neutralizing antibody response,[11,12] the observations above stimulated a systematic analysis of IGHV2-5/IGLV2-14-encoded RBD antibodies to SARS-CoV-2.

RESULTS

Collection of IGHV2-5/IGLV2-14-encoded RBD antibodies

In our previous study, we assembled a dataset of ∼8,000 antibodies to SARS-CoV-2 spike (S) protein.[13] This dataset

This is an open access article under the CC BY license (http://creativecommons.org/licenses/by/4.0/).

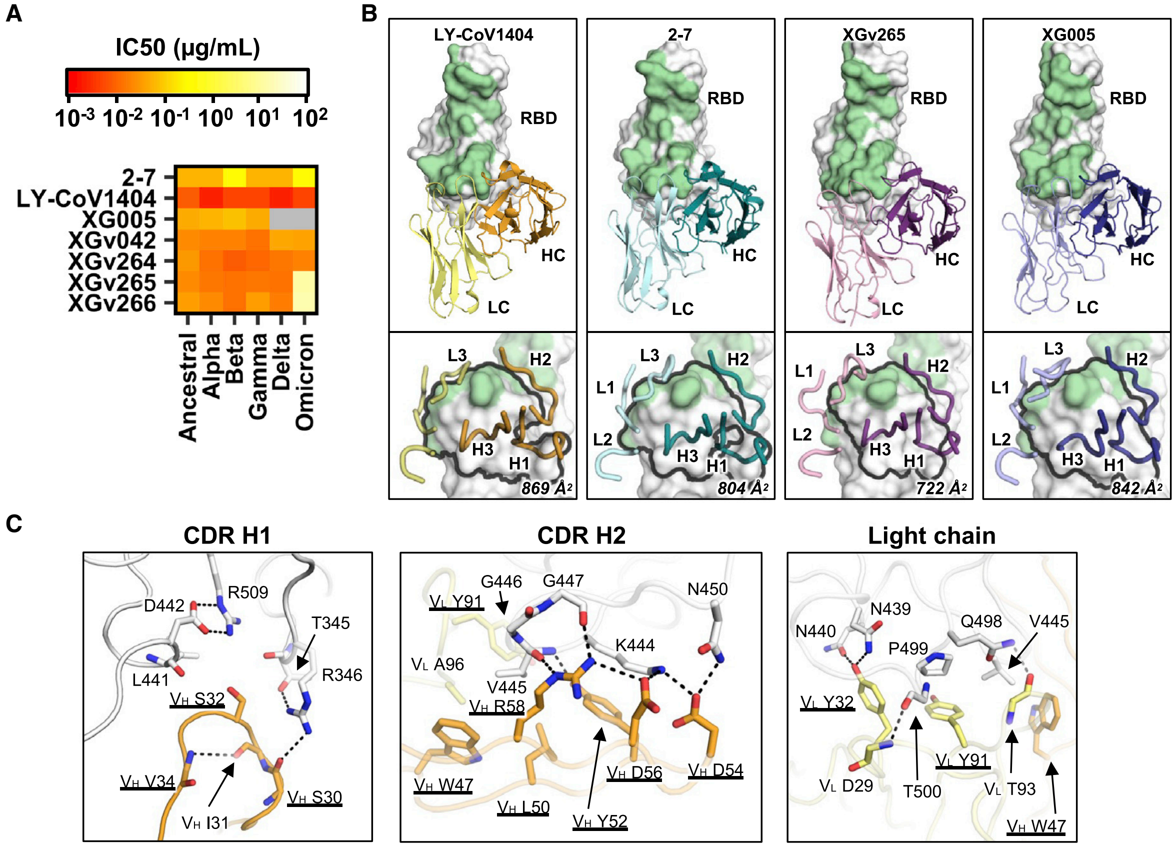

Figure 1. IGHV2-5/IGLV2-14 is a public antibody response

(A) The half-maximal inhibitory concentration (IC_{50}) of IGHV2-5/IGLV2-14-encoded RBD antibodies against different SARS-CoV-2 VOCs in pseudovirus assays. Data were taken from previous studies.[7,19–21] Gray indicates data not available.

(B) Four IGHV2-5/IGLV2-14-encoded RBD antibodies have structure information available. Their binding modes to RBD (white surface) are compared. Top panels: heavy chain (HC) and light chain (LC) of each antibody are shown. Bottom panels: zoom-in views with six CDR loops of each antibody shown. LY-CoV1404, PDB: 7MMO.[7] 2-7, PDB: 7LSS.[22] XGv265, PDB: 7WEE.[19] XG005, PDB: 7V26.[21] ACE2-binding site[42] is in green. Epitope for each antibody is indicated by the black outline in the bottom panels. Buried surface area was calculated by PISA[43] and is shown at the bottom right.

(C) Key interactions between LY-CoV1404 and RBD are shown. Hydrogen bonds and salt bridges are represented by black dashed lines. All germline-encoded residues are underlined. HC is in orange, LC in yellow, and RBD is in white.

See also Figure S1 and Table S1.

contains seven IGHV2-5/IGLV2-14-encoded RBD antibodies, including LY-CoV1404, from six different donors.[7,14–18] In addition, four additional IGHV2-5/IGLV2-14-encoded RBD antibodies were reported in a recent study.[19] Our analysis here is therefore based on a total of 11 IGHV2-5/IGLV2-14-encoded RBD antibodies from at least seven donors. Three of these 11 antibodies have available information for the complete nucleotide sequence, nine have complete amino acid sequence information, 10 have amino acid sequence information for the complementarity-determining regions (CDRs) H3 and L3, and four have structure information. Neutralizing data from previous studies have demonstrated that these IGHV2-5/IGLV2-14-encoded RBD antibodies have high cross-neutralizing activity,[7,19–21] some of which remain potent against Omicron (Figure 1A). Previous studies have also shown that they compete with ACE2 for RBD binding[7,19,21,22] (Figure S1).

Germline-encoded residues in IGHV2-5/IGLV2-14 are important for RBD binding

Next, we performed a structural analysis to uncover the sequence determinants of IGHV2-5/IGLV2-14-encoded antibodies for RBD engagement. For antibody residues, the Kabat numbering scheme is used unless otherwise stated. All four IGHV2-5/IGLV2-14-encoded RBD antibodies with available structural information exhibit the same binding mode to the RBD (Figure 1B). As observed in LY-CoV1404, most amino acid side chains in the paratope are germline encoded and form key interactions with the RBD (Figure 1C). For example, while most CDR H1 contacts are mediated by its main chain, the side chain of germline-encoded V_H S32 in CDR H1 fits into a polar pocket in the RBD. In addition, germline-encoded V_H Y52, D54, D56, and R58 in CDR H2 form an extensive network of H-bonds and electrostatic interactions with the RBD. Furthermore, two key paratope residues in the light chain V_L Y32 and

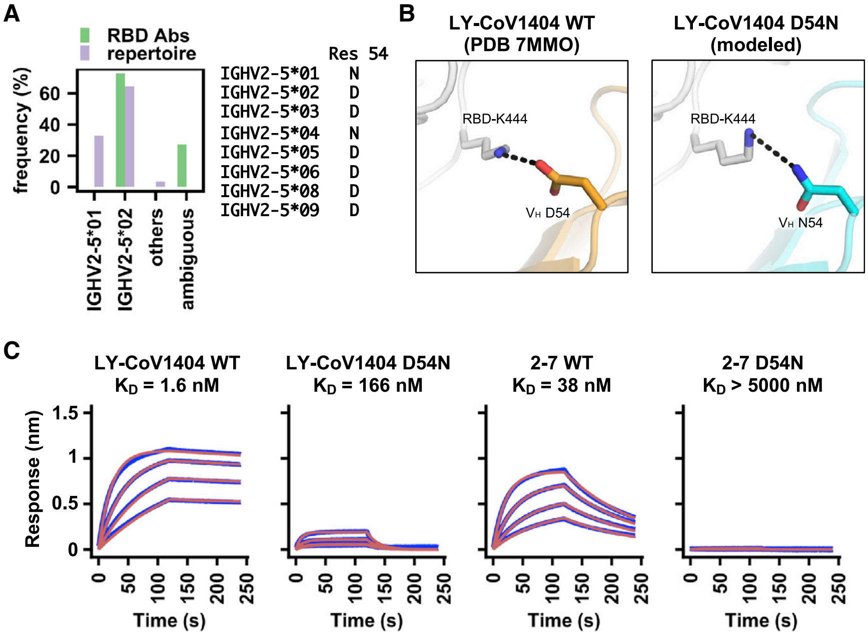

Figure 2. IGHV2-5/IGLV2-14-encoded RBD antibodies have a strong allelic preference

(A) IGHV allele usage of the 11 IGHV2-5/IGLV2-14-encoded RBD antibodies (RBD Abs) is compared with that of IGHV2-5-encoded Abs in published repertoire sequencing datasets from 13 healthy donors.[23,24] The amino acid identity at residue 54 of each IGHV2-5 allele is indicated.

(B) Interactions between the Ab residue 54 and RBD-K444. The RBD is shown in white, wild-type LY-CoV1404 (left, PDB: 7MMO) in orange, and LY-CoV1404 D54N (right, modeled) in cyan. A salt bridge and H-bond are shown as black dashed lines.

(C) Binding kinetics of different Fabs against recombinant SARS-CoV-2 RBD were measured by biolayer interferometry (BLI). The y axis represents the response. Blue lines represent the response curve, and red lines represent a 1:1 binding model. Binding kinetics were measured for four Fab concentrations.

See also Tables S1 and S2.

Y91 are also germline encoded. V_L Y32 H-bonds with RBD N439, whereas V_L Y91 stacks with RBD P499. Both N439 and P499 are conserved among all VOCs to date. These observations demonstrate that the RBD-binding determinants are encoded in the germline sequences of IGHV2-5 and IGLV2-14. Consistently, several IGHV2-5/IGLV2-14-encoded RBD antibodies have very few somatic hypermutations (SHMs) (Table S1). For example, S24-223 has only one SHM, and COV2-2268 and 2-7 have only four each. Of note, none of their SHMs overlap.

IGHV2-5/IGLV2-14-encoded RBD antibodies have an allelic preference

Additional sequence analysis indicated that IGHV2-5/IGLV2-14-encoded RBD antibodies had a strong allelic preference toward IGHV2-5*02. Eight out of 11 IGHV2-5/IGLV2-14-encoded RBD antibodies could be assigned to IGHV2-5*02, while the allele usage for the other three was ambiguous (Figure 2A; Table S1). In contrast, analysis of the B cell repertoire in 13 healthy donors[23,24] showed that alleles IGHV2-5*01 and IGHV2-5*02 were both commonly used, with a frequency of 33% and 64%, respectively, among all IGHV2-5 antibodies (Figure 2A; Table S2).

The lack of IGHV2-5*01 among IGHV2-5/IGLV2-14-encoded RBD antibodies is likely due to an allelic polymorphism at residue 54. IGHV2-5*01 and IGHV2-5*02 have Asn and Asp, respectively, at residue 54. V_H D54 in IGHV2-5/IGLV2-14-encoded RBD antibodies plays an important role in RBD binding through a salt bridge with RBD K444 and an H-bond with RBD N450 (Figure 1C). As demonstrated by structural modeling using Rosetta,[25–27] replacing the Asp at V_H residue 54 by Asn would convert the salt bridge with RBD K444 to an H-bond, which weakened the binding energy by 3.2 Rosetta energy unit (Figure 2B). This observation was further validated by a binding experiment, which showed that mutation D54N weakened the RBD binding affinity (K_D) of LY-CoV1404 and 2-7, both of which are IGHV2-5/IGLV2-14-encoded RBD antibodies (Figures 1A and 1B; Table S1), by at least 100 fold (Figure 2C). Consistently, all eight of the nine IGHV2-5/IGLV2-14-encoded RBD antibodies with sequence information available have an Asp at V_H residue 54, whereas the remaining one has a Glu at V_H residue 54 (Table S1). These findings provide a mechanistic basis for the allelic preference against IGHV2-5*01 despite its prevalence in the human population. Coincidentally, an almost identical observation was observed in an IGHV2-5-encoded HIV antibody, in which V_H D54 results in much stronger binding than V_H N54.[28]

Sequence features of CDR H3 in IGHV2-5/IGLV2-14-encoded RBD antibodies

Lastly, we analyzed the CDR H3 sequences of the IGHV2-5/IGLV2-14-encoded RBD antibodies. Among 10 IGHV2-5/IGLV2-14-encoded RBD antibodies with CDR H3 sequence information available, eight had a CDR H3 length of 11 amino acids (IMGT numbering) and came from at least five patients (Figure 3A). The CDR H3 sequences from these eight antibodies shared a motif HxIxxI or conserved variations of it, including HxIxxL and HxVxxI (Figures 3A and 3B). The HxIxxI motif consisted of V_H H95, I97, and I100 (Kabat numbering) and is uncommon among the CDR H3 sequences of IGHV2-5-encoded antibodies in the human antibody repertoire (Figure 3B). V_H H95, I97, and I100 in the HxIxxI motif play critical roles in stabilizing the loop conformation as well as RBD binding (Figure 3C). V_H H95 forms two intramolecular H-bonds to stabilize the CDR H3 loop. The first H-bond involves the side chain of V_H Y52, which in turn H-bonds with RBD V445 amide nitrogen. The second H-bond involves the backbone carbonyl of V_H I100. In addition, V_H H95 also forms van der Waals interaction with RBD V445. V_H I97 at the tip of the CDR H3 loop inserts into a hydrophobic pocket formed by RBD V445 and P499, as well as the aliphatic portion of RBD N440. V_H I100 helps position V_L Y91 to interact

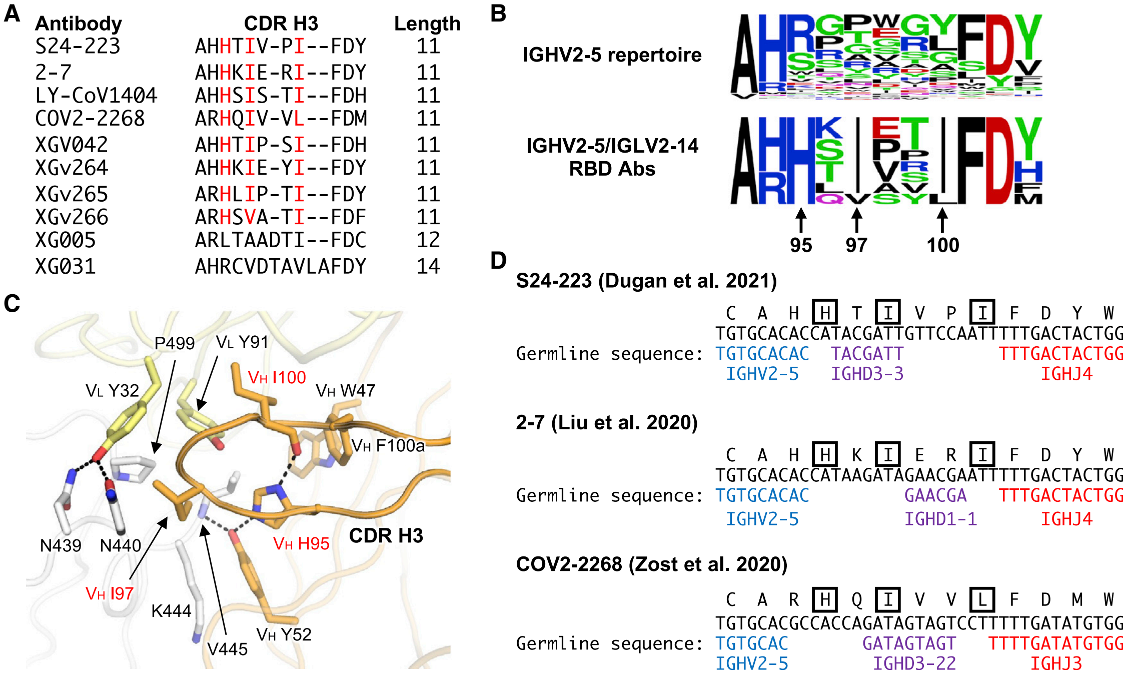

Figure 3. HxIxxI is a common motif in IGHV2-5/IGLV2-14-encoded RBD Abs

(A) CDR H3 sequences (IMGT numbering) from IGHV2-5/IGLV2-14-encoded RBD Abs are aligned. Residues of interest are highlighted in red.

(B) CDR H3 sequences (IMGT numbering) of IGHV2-5/IGLV2-14-encoded RBD Abs and IGHV2-5-encoded Abs in the human Ab repertoire are shown as sequence logos. Only those Abs with a CDR H3 length of 11 amino acids are included in this analysis. Residues of interest are labeled. Sequences of IGHV2-5-encoded Abs in the human Ab repertoire were downloaded from the Observed Antibody Space.[44] A total of 9,197 IGHV2-5-encoded Abs in the human Ab repertoire were analyzed here. Of note, while Kabat numbering was used for the residue position, IMGT numbering was used for defining CDR H3 length.

(C) Interaction between the CDR H3 of LY-CoV1404 and RBD is shown. PDB: 7MMO.[7] Hydrogen bonds are represented by black dashed lines. HC is in orange, LC in yellow, and RBD is in white. Residues of interest are highlighted in red.

(D) Amino acid and nucleotide sequences of the V-D-J junction of three IGHV2-5/IGLV2-14-encoded RBD Abs are shown. Putative germline sequences and segments are indicated. Residues of interest are boxed.

See also Figure S2 and Table S1.

with RBD V445 and P499. As shown by IgBlast analysis,[29] the HxIxxI motif is largely encoded by N-nucleotide addition, although V_H I97 may sometimes be encoded by an IGHD gene (Figure 3D). Of note, while CDR H3 of XG005 has 12 amino acids (Figure 3A), it adopts a similar conformation to those with 11 amino acids (Figure S2). Overall, IGHV2-5/IGLV2-14-encoded RBD antibodies with a CDR H3 length of 11 amino acids have convergent CDR H3 sequences and thus can be classified as a public clonotype.

DISCUSSION

Due to the continuous evolution of SARS-CoV-2 VOCs, identification of cross-neutralizing human monoclonal antibodies has been a global research focus. IGHV1-58/IGKV3-20-encoded RBD antibodies are perhaps the most well-characterized public antibody clonotype that is cross-neutralizing against multiple SARS-CoV-2 VOCs.[3,13,30–33] However, recent studies have shown that many IGHV1-58/IGKV3-20-encoded RBD antibodies have minimal neutralizing activity against Omicron and its sub-lineages due to mutations Q493R and F486V on the RBD.[34–36] In comparison, IGHV2-5/IGLV2-14-encoded RBD antibodies, which mostly retain potency against Omicron and its sub-lineages (Figure 1A),[7,8,19–21,37] have higher neutralization breadth. Since IGHV2-5/IGLV2-14-encoded RBD antibodies are also a public antibody clonotype, they further substantiate the rationale and strategy for development of a more universal COVID-19 vaccine.

Nevertheless, some individuals may have difficulties generating an IGHV2-5/IGLV2-14-encoded RBD antibody response due to the alleles that they possess (Figure 2). Since there is no known copy-number variation for IGHV2-5,[38] each person should carry two copies of IGHV2-5 in the genome. If both copies are IGHV2-5*01 allele, the person may not have the suitable B cell germline clone to produce an IGHV2-5/IGLV2-14-encoded RBD antibody response. In fact, donor 112 in the 13 healthy donors that were analyzed in this study is very likely to be IGHV2-5*01 homozygous since 94% of the IGHV2-5-encoded antibodies were assigned to IGHV2-5*01 (Table S2). Moreover, the conserved HxIxxI motif in CDR H3 of IGHV2-5/IGLV2-14-encoded RBD antibodies is mostly encoded by random N-nucleotide addition. As a result, B cell germline clones that can produce IGHV2-5/IGLV2-14-encoded RBD antibodies may be relatively rare. These results suggest that the ability to generate IGHV2-5/IGLV2-14-encoded cross-neutralizing antibody response is heterogeneous among different individuals.

CellPress
OPEN ACCESS

While allelic preference has previously been described for neutralizing antibodies to other viruses,[28,39–41] its clinical implications for COVID-19 remain to be fully explored.

Limitations of the study

While structures of four IGHV2-5/IGLV2-14 antibodies in complex with SARS-CoV-2 RBD are available in PDB, only LY-CoV1404 (PDB: 7MMO) had a moderate to high resolution at 2.4 Å.[7] In contrast, the other three structures (2-7, PDB: 7LSS; XGv265, PDB: 7WEE; XG005, PDB: 7V26) had a lower resolution (>3.7 Å).[19,21,22] As a result, structural modeling of V_H D54N was only performed for the LY-CoV1404.

STAR★METHODS

Detailed methods are provided in the online version of this paper and include the following:

- KEY RESOURCES TABLE
- RESOURCE AVAILABILITY
  - Lead contact
  - Materials availability
  - Data and code availability
- EXPERIMENTAL MODELS AND SUBJECT DETAILS
- METHOD DETAILS
  - Collection of antibody information
  - Allele assignment of IGHV2-5/IGLV2-14-encoded RBD antibodies
  - Analysis of allele usage in published antibody repertoire
  - Analysis of CDR H3 sequences
  - Expression and purification of Fabs
  - Expression and purification of RBD
  - Biolayer interferometry binding assay
  - ΔΔG calculation
- QUANTIFICATION AND STATISTICAL ANALYSIS

SUPPLEMENTAL INFORMATION

Supplemental information can be found online at https://doi.org/10.1016/j.celrep.2022.111650.

ACKNOWLEDGMENTS

This work was supported by National Institutes of Health (NIH) DP2 AT011966 (N.C.W.) and R01 AI167910 (N.C.W.), the Michelson Prizes for Human Immunology and Vaccine Research (N.C.W.), Bill and Melinda Gates Foundation INV-004923 (I.A.W.), and a Calmette and Yersin scholarship from the Pasteur International Network Association (H.L.).

AUTHOR CONTRIBUTIONS

M.Y. and N.C.W. conceived and designed the study. All authors performed data analysis. M.Y. and N.C.W. wrote the paper, and all authors reviewed and/or edited the paper.

DECLARATION OF INTERESTS

N.C.W. serves as a consultant for HeliXon.

Received: May 19, 2022
Revised: July 13, 2022
Accepted: October 20, 2022
Published: November 15, 2022

REFERENCES

1. Zhang, Q., Ju, B., Ge, J., Chan, J.F.W., Cheng, L., Wang, R., Huang, W., Fang, M., Chen, P., Zhou, B., et al. (2021). Potent and protective IGHV3-53/3-66 public antibodies and their shared escape mutant on the spike of SARS-CoV-2. Nat. Commun. *12*, 4210. https://doi.org/10.1038/s41467-021-24514-w.

2. Yuan, M., Huang, D., Lee, C.C.D., Wu, N.C., Jackson, A.M., Zhu, X., Liu, H., Peng, L., van Gils, M.J., Sanders, R.W., et al. (2021). Structural and functional ramifications of antigenic drift in recent SARS-CoV-2 variants. Science *373*, 818–823. https://doi.org/10.1126/science.abh1139.

3. Reincke, S.M., Yuan, M., Kornau, H.C., Corman, V.M., van Hoof, S., Sánchez-Sendin, E., Ramberger, M., Yu, W., Hua, Y., Tien, H., et al. (2022). SARS-CoV-2 Beta variant infection elicits potent lineage-specific and cross-reactive antibodies. Science *375*, 782–787. https://doi.org/10.1126/science.abm5835.

4. Zhou, P., Yuan, M., Song, G., Beutler, N., Shaabani, N., Huang, D., He, W.T., Zhu, X., Callaghan, S., Yong, P., et al. (2022). A human antibody reveals a conserved site on beta-coronavirus spike proteins and confers protection against SARS-CoV-2 infection. Sci. Transl. Med. *14*, eabi9215. https://doi.org/10.1126/scitranslmed.abi9215.

5. Pinto, D., Sauer, M.M., Czudnochowski, N., Low, J.S., Tortorici, M.A., Housley, M.P., Noack, J., Walls, A.C., Bowen, J.E., Guarino, B., et al. (2021). Broad betacoronavirus neutralization by a stem helix-specific human antibody. Science *373*, 1109–1116. https://doi.org/10.1126/science.abj3321.

6. Li, W., Chen, Y., Prévost, J., Ullah, I., Lu, M., Gong, S.Y., Tauzin, A., Gasser, R., Vézina, D., Anand, S.P., et al. (2022). Structural basis and mode of action for two broadly neutralizing antibodies against SARS-CoV-2 emerging variants of concern. Cell Rep. *38*, 110210. https://doi.org/10.1016/j.celrep.2021.110210.

7. Westendorf, K., Žentelis, S., Wang, L., Foster, D., Vaillancourt, P., Wiggin, M., Lovett, E., van der Lee, R., Hendle, J., Pustilnik, A., et al. (2022). LY-CoV1404 (bebtelovimab) potently neutralizes SARS-CoV-2 variants. Cell Rep. *39*, 110812. https://doi.org/10.1016/j.celrep.2022.110812.

8. Cao, Y., Yisimayi, A., Jian, F., Song, W., Xiao, T., Wang, L., Du, S., Wang, J., Li, Q., Chen, X., et al. (2022). BA.2.12.1, BA.4 and BA.5 escape antibodies elicited by Omicron infection. Nature *608*, 593–602. https://doi.org/10.1038/s41586-022-04980-y.

9. Kramer, K.J., Johnson, N.V., Shiakolas, A.R., Suryadevara, N., Periasamy, S., Raju, N., Williams, J.K., Wrapp, D., Zost, S.J., Walker, L.M., et al. (2021). Potent neutralization of SARS-CoV-2 variants of concern by an antibody with an uncommon genetic signature and structural mode of spike recognition. Cell Rep. *37*, 109784. https://doi.org/10.1016/j.celrep.2021.109784.

10. Park, Y.J., Pinto, D., Walls, A.C., Liu, Z., De Marco, A., Benigni, F., et al. (2022). Imprinted antibody responses against SARS-CoV-2 Omicron sublineages. Science. https://doi.org/10.1126/science.adc9127.

11. Andreano, E., Paciello, I., Piccini, G., Manganaro, N., Pileri, P., Hyseni, I., Leonardi, M., Pantano, E., Abbiento, V., Benincasa, L., et al. (2021). Hybrid immunity improves B cells and antibodies against SARS-CoV-2 variants. Nature *600*, 530–535. https://doi.org/10.1038/s41586-021-04117-7.

12. Andreano, E., Paciello, I., Pierleoni, G., Piccini, G., Abbiento, V., Antonelli, G., Pileri, P., Manganaro, N., Pantano, E., Maccari, G., et al. (2022). COVID-19 mRNA third dose induces a unique hybrid immunity-like antibody response. Preprint at bioRxiv. https://doi.org/10.1101/2022.05.09.491201.

13. Wang, Y., Yuan, M., Lv, H., Peng, J., Wilson, I.A., and Wu, N.C. (2022). A large-scale systematic survey reveals recurring molecular features of

public antibody responses to SARS-CoV-2. Immunity *55*, 1105–1117.e4. https://doi.org/10.1016/j.immuni.2022.03.019.

14. Dugan, H.L., Stamper, C.T., Li, L., Changrob, S., Asby, N.W., Halfmann, P.J., Zheng, N.Y., Huang, M., Shaw, D.G., Cobb, M.S., et al. (2021). Profiling B cell immunodominance after SARS-CoV-2 infection reveals antibody evolution to non-neutralizing viral targets. Immunity *54*, 1290–1303.e7. https://doi.org/10.1016/j.immuni.2021.05.001.
15. Ju, B., Zhang, Q., Ge, J., Wang, R., Sun, J., Ge, X., Yu, J., Shan, S., Zhou, B., Song, S., et al. (2020). Human neutralizing antibodies elicited by SARS-CoV-2 infection. Nature *584*, 115–119. https://doi.org/10.1038/s41586-020-2380-z.
16. Liu, L., Wang, P., Nair, M.S., Yu, J., Rapp, M., Wang, Q., Luo, Y., Chan, J.F.W., Sahi, V., Figueroa, A., et al. (2020). Potent neutralizing antibodies directed to multiple epitopes on SARS-CoV-2 spike. Nature *584*, 450–456. https://doi.org/10.1038/s41586-020-2571-7.
17. Zhou, Y., Liu, Z., Li, S., Xu, W., Zhang, Q., Silva, I.T., Li, C., Wu, Y., Jiang, Q., Liu, Z., et al. (2021). Enhancement versus neutralization by SARS-CoV-2 antibodies from a convalescent donor associates with distinct epitopes on the RBD. Cell Rep. *34*, 108699. https://doi.org/10.1016/j.celrep.2021.108699.
18. Zost, S.J., Gilchuk, P., Chen, R.E., Case, J.B., Reidy, J.X., Trivette, A., Nargi, R.S., Sutton, R.E., Suryadevara, N., Chen, E.C., et al. (2020). Rapid isolation and profiling of a diverse panel of human monoclonal antibodies targeting the SARS-CoV-2 spike protein. Nat Med *26*, 1422–1427. https://doi.org/10.1101/2020.05.12.091462.
19. Wang, K., Jia, Z., Bao, L., Wang, L., Cao, L., Chi, H., Hu, Y., Li, Q., Zhou, Y., Jiang, Y., et al. (2022). Memory B cell repertoire from triple vaccinees against diverse SARS-CoV-2 variants. Nature *603*, 919–925. https://doi.org/10.1038/s41586-022-04466-x.
20. Liu, L., Iketani, S., Guo, Y., Chan, J.F.W., Wang, M., Liu, L., Luo, Y., Chu, H., Huang, Y., Nair, M.S., et al. (2022). Striking antibody evasion manifested by the Omicron variant of SARS-CoV-2. Nature *602*, 676–681. https://doi.org/10.1038/s41586-021-04388-0.
21. Liu, Z., Xu, W., Chen, Z., Fu, W., Zhan, W., Gao, Y., Zhou, J., Zhou, Y., Wu, J., Wang, Q., et al. (2022). An ultrapotent pan-β-coronavirus lineage B (β-CoV-B) neutralizing antibody locks the receptor-binding domain in closed conformation by targeting its conserved epitope. Protein Cell *13*, 655–675. https://doi.org/10.1007/s13238-021-00871-6.
22. Cerutti, G., Rapp, M., Guo, Y., Bahna, F., Bimela, J., Reddem, E.R., Yu, J., Wang, P., Liu, L., Huang, Y., et al. (2021). .1.1.7 variants by two potent SARS-CoV-2 neutralizing antibodies. Structural basis for accommodation of emerging B.1.351 and B *29*, 655–663.e4. https://doi.org/10.1016/j.str.2021.05.014.
23. Briney, B., Inderbitzin, A., Joyce, C., and Burton, D.R. (2019). Commonality despite exceptional diversity in the baseline human antibody repertoire. Nature *566*, 393–397. https://doi.org/10.1038/s41586-019-0879-y.
24. Soto, C., Bombardi, R.G., Branchizio, A., Kose, N., Matta, P., Sevy, A.M., Sinkovits, R.S., Gilchuk, P., Finn, J.A., and Crowe, J.E., Jr. (2019). High frequency of shared clonotypes in human B cell receptor repertoires. Nature *566*, 398–402. https://doi.org/10.1038/s41586-019-0934-8.
25. Conway, P., Tyka, M.D., DiMaio, F., Konerding, D.E., and Baker, D. (2014). Relaxation of backbone bond geometry improves protein energy landscape modeling. Protein Sci. *23*, 47–55. https://doi.org/10.1002/pro.2389.
26. Kortemme, T., and Baker, D. (2002). A simple physical model for binding energy hot spots in protein-protein complexes. Proc. Natl. Acad. Sci. USA *99*, 14116–14121. https://doi.org/10.1073/pnas.202485799.
27. Kortemme, T., Kim, D.E., and Baker, D. (2004). Computational alanine scanning of protein-protein interfaces. Sci. STKE *2004*, l2, 2004. https://doi.org/10.1126/stke.2192004pl2.
28. Alam, S.M., Liao, H.X., Dennison, S.M., Jaeger, F., Parks, R., Anasti, K., Foulger, A., Donathan, M., Lucas, J., Verkoczy, L., et al. (2011). Differential reactivity of germ line allelic variants of a broadly neutralizing HIV-1 antibody to a gp41 fusion intermediate conformation. J. Virol. *85*, 11725–11731. https://doi.org/10.1128/JVI.05680-11.
29. Ye, J., Ma, N., Madden, T.L., and Ostell, J.M. (2013). IgBLAST: an immunoglobulin variable domain sequence analysis tool. Nucleic Acids Res. *41*, W34–W40. https://doi.org/10.1093/nar/gkt382.
30. Dejnirattisai, W., Zhou, D., Ginn, H.M., Duyvesteyn, H.M.E., Supasa, P., Case, J.B., Zhao, Y., Walter, T.S., Mentzer, A.J., Liu, C., et al. (2021). The antigenic anatomy of SARS-CoV-2 receptor binding domain. Cell *184*, 2183–2200.e22. https://doi.org/10.1016/j.cell.2021.02.032.
31. Robbiani, D.F., Gaebler, C., Muecksch, F., Lorenzi, J.C.C., Wang, Z., Cho, A., Agudelo, M., Barnes, C.O., Gazumyan, A., Finkin, S., et al. (2020). Convergent antibody responses to SARS-CoV-2 in convalescent individuals. Nature *584*, 437–442. https://doi.org/10.1038/s41586-020-2456-9.
32. Tortorici, M.A., Beltramello, M., Lempp, F.A., Pinto, D., Dang, H.V., Rosen, L.E., McCallum, M., Bowen, J., Minola, A., Jaconi, S., et al. (2020). Ultrapotent human antibodies protect against SARS-CoV-2 challenge via multiple mechanisms. Science *370*, 950–957. https://doi.org/10.1126/science.abe3354.
33. Wang, L., Zhou, T., Zhang, Y., Yang, E.S., Schramm, C.A., Shi, W., Pegu, A., Oloniniyi, O.K., Henry, A.R., Darko, S., et al. (2021). Ultrapotent antibodies against diverse and highly transmissible SARS-CoV-2 variants. Science *373*, eabh1766. https://doi.org/10.1126/science.abh1766.
34. Dong, J., Zost, S.J., Greaney, A.J., Starr, T.N., Dingens, A.S., Chen, E.C., Chen, R.E., Case, J.B., Sutton, R.E., Gilchuk, P., et al. (2021). Genetic and structural basis for SARS-CoV-2 variant neutralization by a two-antibody cocktail. Nat. Microbiol. *6*, 1233–1244. https://doi.org/10.1038/s41564-021-00972-2.
35. Starr, T.N., Czudnochowski, N., Liu, Z., Zatta, F., Park, Y.J., Addetia, A., Pinto, D., Beltramello, M., Hernandez, P., Greaney, A.J., et al. (2021). SARS-CoV-2 RBD antibodies that maximize breadth and resistance to escape. Nature *597*, 97–102. https://doi.org/10.1038/s41586-021-03807-6.
36. FDA (2021). Fact Sheet for Healthcare Providers: Emergency Ese Authorization for Evusheld (Tixagevimab Co-packaged with Cilgavimab).
37. Yamasoba, D., Kosugi, Y., Kimura, I., Fujita, S., Uriu, K., Ito, J., and Sato, K.; Genotype to Phenotype Japan G2P-Japan Consortium (2022). Neutralisation sensitivity of SARS-CoV-2 omicron subvariants to therapeutic monoclonal antibodies. Lancet Infect. Dis. *22*, 942–943. https://doi.org/10.1016/S1473-3099(22)00365-6.
38. Lefranc, M.P., and Lefranc, G. (2020). Immunoglobulins or antibodies: IMGT bridging genes, structures and functions. Biomedicines *8*, 319. https://doi.org/10.3390/biomedicines8090319.
39. Ying, T., Prabakaran, P., Du, L., Shi, W., Feng, Y., Wang, Y., Wang, L., Li, W., Jiang, S., Dimitrov, D.S., and Zhou, T. (2015). Junctional and allele-specific residues are critical for MERS-CoV neutralization by an exceptionally potent germline-like antibody. Nat. Commun. *6*, 8223. https://doi.org/10.1038/ncomms9223.
40. Lang, S., Xie, J., Zhu, X., Wu, N.C., Lerner, R.A., and Wilson, I.A. (2017). Antibody 27F3 broadly targets influenza A group 1 and 2 hemagglutinins through a further variation in V_H1-69 antibody orientation on the HA stem. Cell Rep. *20*, 2935–2943. https://doi.org/10.1016/j.celrep.2017.08.084.
41. Avnir, Y., Watson, C.T., Glanville, J., Peterson, E.C., Tallarico, A.S., Bennett, A.S., Qin, K., Fu, Y., Huang, C.Y., Beigel, J.H., et al. (2016). IGHV1-69 polymorphism modulates anti-influenza antibody repertoires, correlates with IGHV utilization shifts and varies by ethnicity. Sci. Rep. *6*, 20842. https://doi.org/10.1038/srep20842.
42. Lan, J., Ge, J., Yu, J., Shan, S., Zhou, H., Fan, S., Zhang, Q., Shi, X., Wang, Q., Zhang, L., and Wang, X. (2020). Structure of the SARS-CoV-2 spike receptor-binding domain bound to the ACE2 receptor. Nature *581*, 215–220. https://doi.org/10.1038/s41586-020-2180-5.

43. Krissinel, E., and Henrick, K. (2007). Inference of macromolecular assemblies from crystalline state. J. Mol. Biol. *372*, 774–797. https://doi.org/10.1016/j.jmb.2007.05.022.

44. Kovaltsuk, A., Leem, J., Kelm, S., Snowden, J., Deane, C.M., and Krawczyk, K. (2018). Observed Antibody Space: a resource for data mining next-generation sequencing of antibody repertoires. J. Immunol. *201*, 2502–2509.

45. Raybould, M.I.J., Kovaltsuk, A., Marks, C., and Deane, C.M. (2021). CoV-AbDab: the coronavirus antibody database. Bioinformatics *37*, 734–735. https://doi.org/10.1093/bioinformatics/btaa739.

46. Guo, Y., Chen, K., Kwong, P.D., Shapiro, L., and Sheng, Z. (2019). cAb-Rep: a database of curated antibody repertoires for exploring antibody diversity and predicting antibody prevalence. Front. Immunol. *10*, 2365. https://doi.org/10.3389/fimmu.2019.02365.

47. Katoh, K., and Standley, D.M. (2013). MAFFT multiple sequence alignment software version 7: improvements in performance and usability. Mol. Biol. Evol. *30*, 772–780. https://doi.org/10.1093/molbev/mst010.

48. Crooks, G.E., Hon, G., Chandonia, J.M., and Brenner, S.E. (2004). WebLogo: a sequence logo generator. Genome Res. *14*, 1188–1190. https://doi.org/10.1101/gr.849004.

49. Wu, N.C., Yuan, M., Bangaru, S., Huang, D., Zhu, X., Lee, C.C.D., Turner, H.L., Peng, L., Yang, L., Burton, D.R., et al. (2020). A natural mutation between SARS-CoV-2 and SARS-CoV determines neutralization by a cross-reactive antibody. PLoS Pathog. *16*, e1009089. https://doi.org/10.1371/journal.ppat.1009089.

50. Yuan, M., Wu, N.C., Zhu, X., Lee, C.C.D., So, R.T.Y., Lv, H., Mok, C.K.P., and Wilson, I.A. (2020). A highly conserved cryptic epitope in the receptor-binding domains of SARS-CoV-2 and SARS-CoV. Science *368*, 630–633. https://doi.org/10.1126/science.abb7269.

51. Rodrigues, J.P.G.L.M., Teixeira, J.M.C., Trellet, M., and Bonvin, A.M.J.J. (2018). pdb-tools: a swiss army knife for molecular structures. F1000Res. *7*, 1961. https://doi.org/10.12688/f1000research.17456.1.

STAR★METHODS

KEY RESOURCES TABLE

REAGENT or RESOURCE	SOURCE	IDENTIFIER
**Chemicals, peptides, and recombinant proteins**		
Sodium chloride (NaCl)	Sigma-Aldrich	Cat# S9888
Concentrated hydrochloric acid (HCl)	Sigma-Aldrich	Cat# H1758
BSA	Sigma-Aldrich	Cat# A9418
Tween 20	Fisher Scientific	Cat# BP337-500
**Critical commercial assays**		
In-Fusion HD Cloning Kit	Takara	Cat# 639647
KOD Hot Start DNA Polymerase	EMD Millipore	Cat# 71086-3
PCR Clean-Up and Gel Extraction Kit	Clontech Laboratories	Cat# 740609.250
QIAprep Spin Miniprep Kit	Qiagen	Cat# 27106
NucleoBond Xtra Maxi	Clontech Laboratories	Cat# 740414.100
**Deposited data**		
Collection of antibody information	This study	Table S1
**Experimental models: Cell lines**		
ExpiCHO cells	Thermo Fisher Scientific	Cat# A29127
Expi293F cells	Thermo Fisher Scientific	Cat# A14527
**Recombinant DNA**		
phCMV3-LY-CoV1404 Fab heavy chain	This study	N/A
phCMV3-LY-CoV1404 Fab light chain	This study	N/A
phCMV3-2-7 Fab heavy chain	This study	N/A
phCMV3-2-7 Fab light chain	This study	N/A
phCMV3-SARS-CoV-2-RBD	(Wu et al., 2020)[49]	N/A
**Software and algorithms**		
Octet analysis software 9.0	Fortebio	N/A
Python	https://www.python.org/	N/A
R	https://www.r-project.org/	N/A
IgBLAST	(Ye et al., 2013)[29]	N/A
WebLogo	(Crooks et al., 2004)[48]	N/A
MAFFT	(Katoh and Standley, 2013)[47]	N/A
Custom scripts	This study	https://doi.org/10.5281/zenodo.7196474
**Other**		
ExpiCHO Expression System Kit	Thermo Fisher Scientific	Cat# A29133
Expi293 Expression System Kit	Thermo Fisher Scientific	Cat# A14635
Phosphate-buffered saline (PBS)	Thermo Fisher Scientific	Cat# 14040133
Ni Sepharose excel resin	Cytiva	Cat# 17371202
CaptureSelect CH1-XL Affinity Matrix	Thermo Fisher Scientific	Cat# 1943462010

RESOURCE AVAILABILITY

Lead contact

Information and requests for resources should be directed to and will be fulfilled by the lead contact, Nicholas C. Wu (nicwu@illinois.edu).

Materials availability

All plasmids generated in this study are available from the Lead Contact without restriction.

Data and code availability

- The assembled dataset IGHV2-5/IGLV2-14-encoded RBD antibodies is in Table S1.
- Custom python scripts for all analyses have been deposited to https://doi.org/10.5281/zenodo.7196474.
- Any additional information required to reanalyze the data reported in this paper is available from the lead contact upon request.

EXPERIMENTAL MODELS AND SUBJECT DETAILS

ExpiCHO cells (Chinese hamster ovary cells, female) and Expi293F cells (human embryonic kidney cells, female) were maintained in ExpiCHO expression medium and Expi293 expression medium, respectively, at 37°C with 8% CO_2 according to the manufacturer's instructions (Thermo Fisher Scientific).

METHOD DETAILS

Collection of antibody information

The information on antibodies S24-223, P2B-1E4, 2-7, LY-CoV1404, XG005, XG031, and COV2-2268 were compiled in our previous study,[13] whereas the information on XGv042, XGv264, XGv265, and XGv266 were compiled in CoV-AbDab.[45] Neutralization data of each monoclonal antibody were collected from the original papers (Table S1). Somatic hypermutations were identified by IgBlast.[29]

Allele assignment of IGHV2-5/IGLV2-14-encoded RBD antibodies

For antibodies P2B-1E4, XG005, and XG031, the allele information was obtained from the original publications.[15,17] For other antibodies, IgBlast was used to assign the allele of each antibody.[29] Nucleotide sequence, if available, was used as input for IgBlast. Otherwise, protein sequence was used. If an antibody showed equally likely to be encoded by two or more alleles, the allele assignment would be classified as "ambiguous". All "ambiguous" allele assignments in this study came from antibodies that do not have nucleotide sequence information available, namely XGv264, XGv265, and XGv266. Of note, while IgBlast showed that XGv266 was equally likely to be encoded by IGHV2-5*01 and IGHV2-5*02, we postulated that XGv266 should be assigned to IGHV2-5*02 at the nucleotide level. Specifically, XGv266 had a Glu at V_H residue 54, which was one nucleotide change from the Asp codon used (IGHV2-5*02) but two nucleotides away from Asn (IGHV2-5*01). However, IgBlast did not utilize codon information for allele assignment when the amino acid sequence was used as input.

Analysis of allele usage in published antibody repertoire

Published antibody repertoire sequencing datasets from 13 healthy donors[23,24] were downloaded from cAb-Rep.[46] Putative germline gene alleles for each antibody sequence in these repertoire sequencing datasets from healthy donors were identified by IgBLAST.[29]

Analysis of CDR H3 sequences

Sequence alignment was performed using MAFFT.[47] Antibody sequences in the human antibody repertoire were downloaded from the Observed Antibody Space.[44] IGHV2-5 antibodies as well as their CDR H3 sequences were identified using IgBLAST.[29] Sequence logos were generated by WebLogo.[48] Putative germline sequences and segments in the V-D-J junctions were identified by IgBLAST.[29]

Expression and purification of Fabs

The heavy and light chains were cloned into phCMV3. The plasmids were transiently co-transfected into ExpiCHO cells at a ratio of 2:1 (heavy chain:light chain) using ExpiFectamine CHO Reagent (Thermo Fisher Scientific) according to the manufacturer's instructions. The supernatant was collected at 10 days post-transfection. The IgGs and Fabs were purified with a CaptureSelect CH1-XL Affinity Matrix (Thermo Fisher Scientific) followed by size exclusion chromatography.

Expression and purification of RBD

The receptor-binding domain (RBD) (residues 319–541) of the SARS-CoV-2 spike (S) protein (GenBank: QHD43416.1) was previously cloned into phCMV3 vector and fused with a C-terminal His_6 tag.[49] The plasmids were transiently transfected into Expi293F cells using ExpiFectamine 293 reagent (Thermo Fisher Scientific) according to the manufacturer's instructions. The supernatant was collected at 7 d posttransfection. The His_6-tagged proteins were then purified with Ni Sepharose Excel protein purification resin (Cytiva) followed by size exclusion chromatography.

Biolayer interferometry binding assay

Binding assays were performed by biolayer interferometry (BLI) using an Octet Red instrument (ForteBio) as described previously.[50] Briefly, His_6-tagged wild-type RBD protein at 20 μg/ml in 1x kinetics buffer (1x PBS, pH 7.4, 0.01% BSA and 0.002% Tween 20) was loaded onto Ni-NTA biosensors and incubated with 200 nM, 100 nM, 50 nM, and 25 nM of Fabs. The assay consisted of five steps: 1) baseline: 60 s with 1x kinetics buffer; 2) loading: 180 s with His_6-tagged RBD protein; 3) baseline: 60 s with 1x kinetics buffer; 4) association: 120 s with Fabs; and 5) dissociation: 120 s with 1x kinetics buffer. For estimating the exact K_D, a 1:1 binding model was used.

ΔΔG calculation

Our ΔΔG calculation was based on the structure of SARS-CoV-2 S bound to LY-CoV1404 (PDB ID: 7MMO).[7] Pyroglutamic acid (PCA) and 2-acetamido-2-deoxy-β-D-glucopyranose (NAG) were removed using PyMOL (Schrödinger). Coordinates for one heavy chain and the RBD that interacted with it (amino acid residues 334–527) were extracted using PyMOL. The resulting PDB file was then renumbered using the "pdb_reres.py" script in pdb-tools.[51] The ΔΔG between WT and the D54N mutant heavy chain was calculated using Rosetta (Rosetta Commons). A constraint file was first generated, and the global structure was relaxed using the 'relax' application.[25] Out of the thirty poses, the pose with the lowest score was used for ΔΔG calculation.[26,27] One-hundred poses were generated for ΔΔG calculation and the ΔG values of WT and mutant antibodies were obtained from the lowest-scoring pose. In this case:

$$\Delta G = \Delta G_{antibody/RBD\ complex} - (\Delta G_{antibody} + \Delta G_{RBD}).$$

ΔΔG was calculated as $\Delta G_{mutant} - \Delta G_{WT}$. A positive ΔΔG value suggests that the binding energy of the mutant antibody/RBD complex is greater than that of the WT antibody/RBD complex. The lowest-scoring pose for D54N mutant is shown in Figure 2B.

QUANTIFICATION AND STATISTICAL ANALYSIS

Standard deviation for K_D estimation was computed by Octet analysis software 9.0.

Cell Reports, Volume *41*

Supplemental information

Molecular analysis of a public cross-neutralizing antibody response to SARS-CoV-2

Meng Yuan, Yiquan Wang, Huibin Lv, Timothy J.C. Tan, Ian A. Wilson, and Nicholas C. Wu

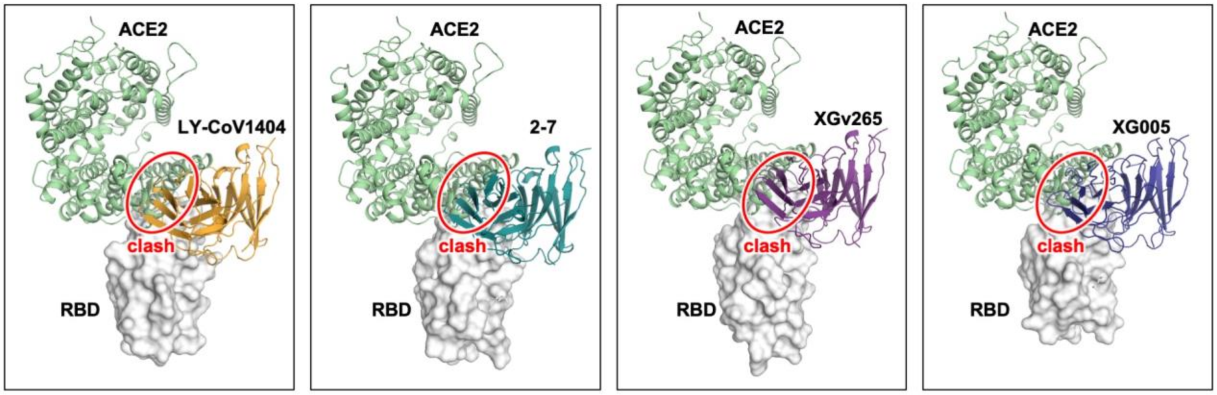

Figure S1. IGHV2-5/IGLV2-14 antibodies would clash with ACE2 binding, Related to Figure 1. Structures of antibody/RBD complexes are superimposed onto the RBD/ACE2 complex structure (PDB 6M0J)[42]. RBD is represented by a white surface. ACE2 is shown as green cartoon. LY-CoV1404: PDB 7MMO[7]. 2-7: PDB 7LSS[22]. XGv265: PDB 7WEE[19]. XG005: PDB 7V26[21].

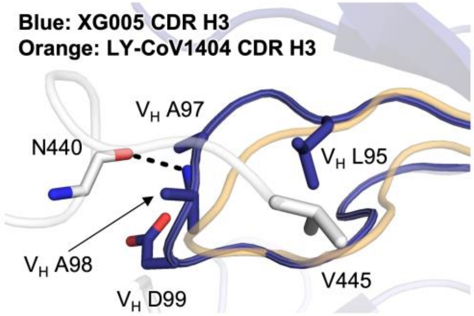

Figure S2. Interactions between RBD and CDR H3 of XG005, Related to Figure 3. A cryoEM structure of SARS-CoV-2 spike protein in complex with XG005 (PDB 7V26) that was reported in a previous study[21] is shown. A hydrogen bond between XG005 and the RBD is represented by a black dashed line. The CDR H3 of LY-CoV1404 (PDB 7MMO)[7] is also shown here as a transparent orange cartoon to demonstrate the relative positions of the CDR H3 loops from these two antibodies after superimposition.
